# Safety and Efficacy of Dexmedetomidine as an Adjuvant in Epidural Anesthesia for Labor Analgesia: A Narrative Review

**DOI:** 10.3390/medsci14010144

**Published:** 2026-03-18

**Authors:** Josephine M. Feeney, Seth J. Duet, Cailyn B. Jones, Anthony J. Baffi, Sandy Rayes Elmalakh, Kristin Nicole Bembenick, Sahar Shekoohi, Shahab Ahmadzadeh

**Affiliations:** 1 School of Medicine, Louisiana State University Health Sciences Center at Shreveport, Shreveport, LA 71103, USA; jmf003@lsuhs.edu (J.M.F.); sdu001@lsuhs.edu (S.J.D.); cbj003@lsuhs.edu (C.B.J.); 2 Department of Anesthesiology, Louisiana State University Health Sciences Center at Shreveport, Shreveport, LA 71103, USA

**Keywords:** dexmedetomidine, opioid-sparing effects, labor analgesia, epidural anesthesia, α_2_ agonist

## Abstract

Effective pain management during labor must balance adequate maternal pain relief with preservation of maternal participation and fetal safety. Epidural anesthesia remains the gold standard for labor analgesia. However, commonly used local anesthetics and opioid adjuvants are associated with adverse effects that include nausea, pruritus, urinary retention, and prolonged labor. Dexmedetomidine, a highly selective α_2_ agonist, does not carry the same risks for misuse and abuse as opioids do and may be a promising non-opioid adjuvant for epidural labor analgesia due to its analgesic, anxiolytic, and opioid-sparing properties. Furthermore, dexmedetomidine has unique pharmacodynamic effects, including preserving maternal consciousness while providing adequate analgesia. This combination of consciousness preservation and sufficient analgesia suggests dexmedetomidine may be a promising pharmaceutic for epidural anesthesia. In addition to preserving maternal consciousness, dexmedetomidine does not appear to cause a clinically significant increase in the motor blockade. Although epidural analgesia is known to prolong labor in nulliparous and multiparous patients, the use of dexmedetomidine as an epidural adjuvant does not have a significant effect on labor duration in available trials. Across studies, dexmedetomidine does not have deleterious outcomes for neonates, measured using the neonatal Apgar score. Although dexmedetomidine is not currently FDA-approved for epidural labor analgesia, existing evidence from available trials suggests its safety and efficacy as an opioid-sparing adjuvant. This narrative review aims to highlight the current state of knowledge of dexmedetomidine’s pharmacology, efficacy, analgesic ability, and side effects.

## 1. Introduction

Modern anesthesia was pioneered by Seishu Hanaoka in 1804 [1]. However, it was not until William Morton’s 1846 demonstration of using ether for anesthesia that this was popularized for pain-free surgeries [2]. In 1847, obstetrician James Young Simpson extended anesthesia’s use to childbirth [3].

As anesthesia use in obstetrics grew, the goals of pain management during labor began to diverge from those of surgical anesthesia. Labor analgesia aims to relieve pain while preserving maternal consciousness so that the pregnant parent can actively participate in childbirth. Additionally, labor analgesia must maintain fetal safety by minimizing exposure to anesthetic agents and maintaining adequate uteroplacental perfusion. Pain management during labor is further complicated by pregnancy-related physiological changes, such as increased blood pressure (BP) [4].

Epidural anesthesia is the gold standard for labor analgesia, providing effective pain relief during labor while preserving maternal alertness [5]. This technique involves injecting local anesthetics into the epidural space outside the dura mater surrounding the spinal cord and nerve roots. While highly effective, epidural anesthesia can prolong labor and may cause side effects including nausea, vomiting, pruritus, shivering, and hypotension [6].

Typical local anesthetics, such as ropivacaine and bupivacaine, used in epidural anesthesia carry potential risks, including fetal bradycardia. Local anesthetics carry the potential risk of causing a clinically significant increase in the motor blockade, which may reduce maternal ability to participate in pushing during epidural anesthesia, without directly affecting uterine smooth muscle contractions [7,8]. A dense motor blockade can hinder maternal participation in the second stage of labor by interfering with skeletal muscles for pushing, especially the abdominal muscles [9,10]. This underscores the need for appropriate dosing that balances pain relief with maternal ability to actively participate in childbirth, the key tradeoff in labor analgesia.

Epidural analgesia using ropivacaine or bupivacaine may also cause maternal hypotension, urinary retention, and prolong the second stage of labor [11]. There is a significant interest in adjunctive therapies that reduce anesthetic requirements. Combining adjunctive therapies to local anesthetics can enhance analgesic efficacy, providing more intense and longer-lasting pain relief [12]. Typically, opioids are used for this purpose. However, opioids carry serious risks of misuse and abuse. Moreover, opioids are frequently associated with postoperative nausea, vomiting, and pruritus, or itchiness [13].

Dexmedetomidine, a relatively new α_2_ agonist, was FDA-approved in 1999 for sedation in mechanically ventilated and non-intubated patients [14]. In 2022, it was also FDA-approved for treating schizophrenia- and bipolar-disorder-associated acute agitation [15].

In the present investigation, we summarize the safety and efficacy of dexmedetomidine as an adjuvant in epidural anesthesia for maternal pain relief during childbirth.

## 2. Methods

### 2.1. Selection of Papers

A PubMed search for this narrative review was conducted using the key words “dexmedetomidine,” “epidural anesthesia,” and “adjuvant.” The literature search was conducted in January 2026 for articles published in English. Articles published up to January 2026 were considered. Initial search results were screened by title and abstract, and potentially relevant studies were selected for full-text review based on their relevance to adjuvant dexmedetomidine in epidural anesthesia for labor analgesia. Papers that were neither randomized controlled trials nor observational studies were excluded. Papers that utilized adjuvant dexmedetomidine administered via another route of administration other than through an epidural were also excluded. Full texts of potentially relevant articles were then reviewed to determine whether they specifically investigated dexmedetomidine as an adjunctive therapy to epidural anesthesia for pain relief in women in active labor. Five studies met the inclusion criteria. The number of papers included in this review was limited by the number of qualifying papers published. High-quality secondary syntheses, including meta-analyses and systematic reviews, were utilized for background information, cross-checking, and safety considerations relevant to the off-label use of dexmedetomidine.

### 2.2. Limitations

The number of papers included in this review was limited by the number of qualifying papers published. Despite increasing interest in epidural dexmedetomidine, clinical studies that specifically evaluate epidural administration during labor are limited. Strict application of inclusion criteria (human labor analgesia, epidural route of administration) yielded a small number of eligible trials. Four studies were single-center, randomized controlled trials. One study utilized an observational design, which is limited by the lack of random assignment and blinding. This narrative review is limited by selection bias. Additionally, there was heterogeneity in how the studies assessed neonatal and maternal safety. For example, one study only assessed neonatal safety using neonatal Apgar scores and umbilical artery pH. Another study assessed neonatal safety using Apgar scores recorded at 1, 5, and 10 min; umbilical artery pH, pCO_2_, and pO_2_; and fetal heart rate. None of the studies utilized reported NICU admission. Moreover, the studies lacked homogeneity in which effects were included in adverse effects as well as at what time intervals these effects were monitored.

## 3. Dexmedetomidine Pharmacodynamics

### 3.1. Analgesic Effects

Dexmedetomidine is a highly selective α_2_ adrenergic receptor agonist [16]. In fact, dexmedetomidine has a 1620:1 selectivity for the α_2_ receptor over the α_1_ receptor, eight times more selective than that of clonidine, another α_2_ agonist, at 220:1. Dexmedetomidine’s higher selectivity for α_2_ receptors may be the cause of its fewer side effects, especially relating to hemodynamic instability, compared to clonidine [17,18]. Dexmedetomidine induces sedation without loss of responsiveness to verbal stimuli [19].

Dexmedetomidine produces analgesia by inhibiting pain transmission at various sites in the pathway. Dexmedetomidine primarily agonizes α_2_ receptors in the dorsal horn. This inhibits the release of substance P, a nociceptive neurotransmitter [20].

Epidural anesthesia is performed by injecting local anesthetics into the epidural space of the spine at levels L3–L4 or L4–L5. Dexmedetomidine is only used as an adjuvant and is never a replacement for local anesthetics in epidural anesthesia.

### 3.2. Epidural Mechanism of Labor Analgesia

**Figure 1:**
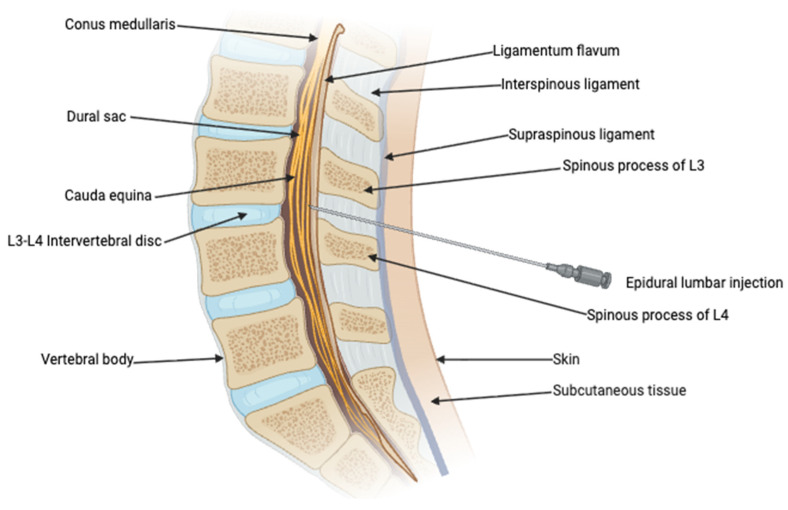
Midsagittal view of epidural lumbar injection. Created with BioRender.com.

Epidural anesthesia is a method of pain control in which local anesthetics are injected into the epidural space in the vertebral canal. The epidural space is located just outside the dura mater and is filled with blood vessels, connective tissue, and fat. Epidural anesthesia has several advantages, including allowing several different types of local anesthetics to be used as well as the type of administration, continuous or intermittent. This enables clinicians to provide tailored pain relief that meets each patient’s needs [21]. The route of administration of an epidural injection is illustrated in Figure 1.

## 4. Dexmedetomidine Pharmacokinetics

Dexmedetomidine is extensively metabolized in the liver and undergoes N-glucuronidation and hydroxylated by CYP2A6 [22]. As a result, hepatic impairment reduces dexmedetomidine clearance, so dexmedetomidine dose must be adjusted in these patients. Decreased cardiac output also decreases dexmedetomidine clearance because lower cardiac output results in a decreased blood supply to the liver. This drug is 94% bound to protein in the blood and has a high volume of distribution. The clearance is 36–42 L/h in adult patients [22]. In sheep, dexmedetomidine has rapid absorption into cerebrospinal fluid when administered via epidural [23].

## 5. Epidural Dexmedetomidine Efficacy in Labor Analgesia

### 5.1. Analgesic Efficacy

In epidural anesthesia for lower limb surgery, dexmedetomidine appeared to reduce the time to onset of sensory blockade, sensation, and analgesia and did not appear to cause a clinically significant increase in the motor blockade when used as an adjuvant to ropivacaine compared to nalbuphine, a synthetic opioid, and clonidine. Dexmedetomidine also increased duration of the sensory blockade [12].

Our primary focus is dexmedetomidine’s efficacy as an adjuvant to epidural anesthesia for labor analgesia. Table 1 summarizes the results of 5 independent studies evaluating this application. Across these studies, dexmedetomidine appeared to decrease visual analog scale (VAS) scores of pain without increasing adverse effects. One randomized, controlled trial (RCT) of 150 women in active labor (parturients) suggested that the women who received dexmedetomidine had a shorter labor duration (608.2 versus 530.4 min, *p* < 0.05), lower VAS scores in the second and third expulsive stages of labor (3.7 versus 2.2, 1.2 versus 0.7, *p* < 0.05), and lower serum levels of IL-6 and TNF-α at delivery and 24 h post-delivery (IL-6: 115.5 versus 106.3 pg/mL, 120.6 versus 108.5 pg/mL, respectively; TNF-α: 8.1 versus 7.4 fmol/mL, 8.3 versus 8.0 fmol/mL, respectively; *p* < 0.05). Both groups received 0.1% ropivacaine. The experimental group (n = 75) additionally received 0.5 µg/mL dexmedetomidine. Analgesia was administered via a PCEA pump with an initial loading dose of 10 mL, followed by a continuous background of 10 mL/h (5 µg/h) and a patient-controlled bolus of 5 mL with a 20 min lockout interval (2.5 μg per demand). There was no significant difference in the decrease in heart rate (HR) and mean arterial pressure (MAP), neonatal Apgar scores, amount of postpartum hemorrhage, and side effects between the two groups. Dexmedetomidine did not significantly increase adverse effects, defined as pruritus, vomiting, nausea, bradycardia, and hypotension. This study found that dexmedetomidine can enhance uterine contraction frequency, decreasing the duration of the second and third stages of labor [24]. However, this study was at a single center, which limits its generalizability to all women in active labor treated with dexmedetomidine as an adjuvant to epidural anesthesia. Additionally, this study excluded women whose fetuses showed signs of fetal distress or cephalopelvic disproportion, women who had given birth before, women with bradycardia, and women who had contraindications to spinal anesthesia. Included women in this study were between the ages of 20 and 35 and nulliparous. Finally, this study did not assess fetal heart rate nor umbilical artery pH, two important parameters for assessing neonatal safety. Due to possible selection bias, this study has a low concern of risk of bias.

Dexmedetomidine can cause hemodynamic instability in some cases. In an RCT of 80 parturients, dexmedetomidine significantly decreased maternal HR, diastolic BP, systolic BP, and VAS scores by a statistically significant amount. However, the decrease in each of these parameters was not clinically significant. Both groups (n = 40) received continuous 0.125% ropivacaine. The experimental group also received 0.5 µg/kg dexmedetomidine in the loading bolus. There was no significant difference in neonatal Apgar scores, duration of first two stages of labor, intensity of maternal sedation, or incidence between the two groups. The hypotension caused by dexmedetomidine had no clinically significant effect on the parturient nor the neonate [25]. This study suggests that dexmedetomidine did not increase adverse effects compared to ropivacaine alone, defined as nausea, vomiting, and maternal sedation. However, this study was at a single center, which limits its generalizability to other populations. Additionally, this study excluded patients with obesity, preeclampsia, fetal macrosomia, and a prior history of complications related to anesthesia. The study only included healthy nulliparous parturient women from 22 to 35 years old. Additionally, this study only recorded Apgar scores and umbilical artery pH to assess the health of the fetus. Therefore, it is unclear whether dexmedetomidine does not cause harmful effects on the fetus according to this study. Due to possible selection and reporting bias, this study has some concerns for risk of bias.

### 5.2. Opioid-Sparing Effects

While dexmedetomidine can cause hemodynamic instability in some cases, it appears to be markedly reduced compared to opioids. Additionally, some studies suggest that adjunctive dexmedetomidine can not only maintain but improve the level of analgesia with a lower amount of ropivacaine [26]. In an RCT of 160 parturients by Cheng et al., subjects in the two dexmedetomidine groups had fewer adverse effects, specifically urinary retention, than subjects in the two sufentanil groups. However, there was bradycardia (n = 6), pruritus (n = 1), vomiting (n = 2), and urinary retention at 6 h (n = 7) across both dexmedetomidine groups. All groups received ropivacaine (0.125% or 0.08%). Sufentanil groups received 0.5 µg/mL sufentanil. Dexmedetomidine groups received 0.5 µg/mL dexmedetomidine with an infusion pump with a 10 mL loading dose, followed by 8 mL/h background infusion. PCEA was 8 mL (4 µg) with a 30 min lockout interval (4 µg/h). The sufentanil groups had significantly decreased HR compared to the dexmedetomidine groups at both concentrations of ropivacaine (0.125% and 0.08% of ropivacaine, respectively: 82.13 versus 89.48 and 80.35 versus 81.42 beats/min, *p* < 0.05). At the same time, the dexmedetomidine groups had significantly lower VAS scores across both concentrations of ropivacaine (0.125% and 0.08% of ropivacaine, respectively: 2.19 versus 1.14, 4.14 versus 2.21, *p* < 0.05). At 2 h postpartum, the sufentanil group receiving the lower dose of ropivacaine still had a VAS score of 2.13 while both dexmedetomidine groups had VAS scores of 0 (*p* < 0.05). This study suggests that VAS scores are significantly lower using dexmedetomidine as an adjuvant instead of opioids, even at lower doses of local anesthetics [26]. Fetal safety was assessed through fetal heart rate; umbilical cord arterial blood pH, pCO_2_, and pO_2_, and Apgar scores at 1, 5, and 10 min. However, this study is limited in its generalizability because it was conducted at a single center and excluded multiparous women; women with severe liver, kidney, heart, and lung diseases; and parturient women with preeclampsia. This study only included women 20 to 35 years old with normal platelet count and coagulation function. This study also defined adverse effects as hypotension, respiratory depression, bradycardia, pruritus, nausea, vomiting, and urinary retention. Due to selection bias, this study has low concern for risk of bias.

A controlled, observational study by Selim et al. of 110 parturients found that dexmedetomidine decreased VAS scores, time to onset of analgesia (5.9 vs. 9.1 min, *p* < 0.05), nausea, and pruritus. Dexmedetomidine increased the duration of analgesia (155.6 vs. 129 min, *p* < 0.05). While there was reduced uterine blood flow in the two groups receiving anesthesia, this did not result in neonatal acidosis or low Apgar scores at birth. Two groups received 12 mL of 0.25% bupivacaine. Of those, one group received 1 µg/kg dexmedetomidine, and the other received 1 µg/kg fentanyl. Both the dexmedetomidine and fentanyl were diluted in 5 mL saline. The control group did not receive anesthesia. Nausea was significantly higher in the group receiving fentanyl versus the group receiving dexmedetomidine (20.5% vs. 4.5%, *p* < 0.05). There was no significant difference in Bromage scores between the two groups receiving anesthesia. The Bromage score evaluates the degree of motor blockade caused by local anesthesia [27]. Notably, this study excluded subjects with pregnancy-induced hypertension, cardiovascular disease, hypercoagulation, or fetal distress that required cesarean delivery. Dexmedetomidine did not appear to cause a clinically significant increase in the motor blockade. While both groups receiving anesthesia had higher uterine artery pulsatility index (UtA-PI) scores and umbilical artery pulsatility index (UA-PI) scores, dexmedetomidine did not increase either score compared to fentanyl. Higher UtA-PI and UA-PI scores indicate poor maternal blood supply and fetal resistance, respectively. In all, the results from this study suggest that dexmedetomidine may have similar analgesic efficacy to opioids but lacks some opioid-induced side effects [28]. However, this study is limited because it is a single-center study. Additionally, subjects were excluded if there was fetal distress, rapid labor progress defined as delivery in less than 2 h, preeclampsia, cardiovascular disease, coagulation disorder, or a bleeding disorder. Due to selection bias and the observational study nature of this paper, this study has a high concern for risk of bias.

Some clinical evidence suggests that dexmedetomidine demonstrates more profound analgesic effects to sufentanil in first-stage labor. In an RCT by [29], 80 parturients received either dexmedetomidine or sufentanil as an adjuvant to epidural anesthesia. The parturients receiving dexmedetomidine had significantly lower VAS scores at all points of increasing cervical dilation before labor analgesia. The experimental group received 0.5 µg/mL dexmedetomidine, and the control group received 0.5 µg/mL sufentanil. Both groups received 0.1% ropivacaine. Additionally, there was less shivering, fetal bradycardia, nausea, and vomiting in the dexmedetomidine group, but the decrease was not statistically significant compared to the sufentanil group (2.7%, 8.3%, and 5.5% versus 8.8%, 5.8%, and 8.8%, respectively). This study defined maternal side effects as respiratory depression, hypotension, pruritus, sedation, nausea, vomiting, shivering, and maternal bradycardia. There were less nausea and vomiting (n = 1) and shivering (n = 2) in the dexmedetomidine group. However, there were 3 instances of fetal bradycardia in the dexmedetomidine group, an increase from 2 in the sufentanil group. The dexmedetomidine group also showed lower analgesic consumption (71.5 versus 78.1, *p* < 0.05) [29]. Neonatal Apgar scares and umbilical artery blood gas analysis were utilized to evaluate neonatal safety. This study is limited because it is a single-center study that excluded women with a history of bradycardia and cardiopulmonary disease. Due to selection bias, this study has low concern for risk of bias.

**Table 1:** Safety and efficacy of dexmedetomidine (DEX) in epidural anesthesia for labor analgesia. * The approximated dose of dexmedetomidine in µg/kg/h was calculated assuming an average patient weight of 65 kg based on study participants. This 65 kg weight may not be generalizable to all obstetric populations. Jun et al. defined side effects as pruritus, nausea, vomiting, urinary retention, hypotension, and bradycardia [24]. Zhao et al. defined side effects as nausea, vomiting, and maternal sedation [25]. ^#^ Indicates that the side effect of hypotension was observed in this trial, which was not considered to be a side effect in Jun et al. [24,26]. Cheng et al. defined adverse effects as hypotension, respiratory depression, bradycardia, pruritus, nausea, vomiting, and urinary retention [26]. Selim et al. defined adverse effects as hypotension, nausea, and pruritus [28]. Zhang et al. defined side effects as respiratory depression, hypotension, pruritus, sedation, nausea, vomiting, shivering, and maternal bradycardia [29].

Author (Year), Study Design	Population (n)	Reported Dose (DEX)	Approximated * DEX Dose in µg/kg/h (Infusion) or µg/kg (Bolus)	Control Group	Side Effects/Safety	Key Findings
Jun et al. (2018), RCT [24]	150 parturients, 75 in experimental group	5 µg/kg	0.08 µg/kg/h (5 µg/kg; PCEA: background 10 mL/h; bolus 5 mL, 20 min lockout)	0.1% ropivacaine	No increase in adverse effects	DEX decreased VAS scores, labor duration
Zhao et al. (2017), RCT [25]	80 parturients, 40 in experimental group	0.5 µg/kg	0.5 µg/kg (bolus)	0.125% ropivacaine	No increase in adverse effects ^#^	DEX significantly lowered diastolic and systolic BP, maternal HR, and VAS scores
Cheng et al. (2019), RCT [26]	160 parturients, experimental group (80); 0.125% ropivacaine (40), 0.08% ropivacaine (40)	5 µg/kg	0.08 µg/kg/h (5 µg/kg; PCEA: background 10 mL/h; bolus 8 mL, 30 min lockout)	0.5 µg/mL sufentanil, 0.125% or 0.08% ropivacaine	Maternal bradycardia (6), pruritus (1), vomiting (2)	DEX lowered VAS scores at lower ropivacaine doses compared to sufentanil
Selim et al. (2012), Observational study [28]	110 parturients: 23 in control, 43 in bupivacaine (B)-DEX, 44 in B-FENT	1 µg/kg	1 µg/kg dissolved in 5 mL saline (bolus)	No analgesia in control group; B-FENT group: 1 µg/kg dissolved in 5 mL saline	Hypotension (12), maternal bradycardia (10), nausea (2), vomiting (1), pruritus (1), fetal bradycardia (5)	DEX increased analgesia duration; decreased VAS scores, time to analgesia, and sedation scores
Zhang et al. (2019), RCT [29]	80 parturients, 40 in experimental	3 µg/kg	0.05 µg/kg/h (3 µg/kg; PCEA: background 6 mL/h; bolus 6 mL, 20 min lockout)	0.5 µg/mL sufentanil	Nausea and vomiting (1), fetal bradycardia (3), shivering (2)	DEX lowered VAS scores more than sufentanil

### 5.3. Labor Progress Effects of Dexmedetomidine

Normal labor consists of three stages: cervical dilation (Stage 1), fetal delivery (Stage 2), and placental delivery (Stage 3) [30,31,32]. Labor duration is variable and influenced by parity, anesthesia, and maternal-fetal factors [30,31,32,33]. The second stage of labor has an average duration of one hour but may extend up to four hours with epidural anesthesia [31,32,34].

Prolongation of the second stage may increase the risk of cesarean delivery, uterine bleeding, and neonatal injury [30,35]. Therefore, the impact of adjunctive dexmedetomidine on labor duration warrants evaluation. Recent meta-analyses suggest dexmedetomidine does not significantly prolong any stage of labor [36,37].

### 5.4. Changes in Pregnancy and Their Associated Anesthetic Implications

Pregnancy produces predictable physiologic changes relevant to labor anesthetic management [38]. Maternal blood volume and cardiac output increase significantly, with further transient increases during labor [38,39,40,41]. Systemic vascular resistance decreases. Aortocaval compression in the supine position may reduce venous return and uteroplacental perfusion [38,42]. Pregnancy is a hypercoagulable state with increased fibrinogen and clotting factors [43,44,45,46,47,48,49]. Platelet count and clinical context (rapid sequence induction and aspiration prophylaxis) should be assessed prior to epidural anesthesia placement [38,41].

Cerebral blood flow increases as a result of decreased cerebrovascular resistance [38]. Compression of the dural sac and reduced lumbosacral CSF volume, may influence the epidural spread of local anesthetics due to reduced epidural free space [50,51]. Glomerular filtration rate and renal blood flow increase while plasma albumin decreases, potentially altering drug pharmacokinetics [38,52]. Uteroplacental circulation depends on maternal MAP and uterine vascular resistance. Maternal hypotension and hypoxemia can reduce fetal oxygen delivery [53]. During labor, catecholamine surges may further impact uteroplacental blood flow [54].

## 6. Dexmedetomidine Safety Considerations and Off-Label Neuraxial Use

### 6.1. Maternal Adverse Events

Dexmedetomidine can cause hemodynamic instability, manifesting as transient hypertension, bradycardia, and hypotension [17]. To assess maternal safety in epidural anesthesia, several types of adverse events are recorded, including hypotension, respiratory depression, bradycardia, urinary retention, pruritus, and nausea and vomiting [55]. Maternal hypotension was defined across all 5 studies as a 20% or more decrease in MAP. Maternal bradycardia was defined as less than 60 beats per minute. Respiratory depression was defined as oxygen saturation less than 92% or respiratory rate less than 10 breaths per minute. Assessment intervals of urinary retention varied across studies. Across included studies in dexmedetomidine groups, the incidence of hypotension ranged from 0–27.2%, while respiratory depression was not observed. Bradycardia occurred in 0–22.7% of patients, urinary retention in 1–9%, pruritus ranged in 0–1%, and nausea and vomiting ranged in 0–3%. Across control groups in the included studies, hypotension occurred in 0–18.6%, bradycardia in 0–11.6%, urinary retention in 3–19%, pruritus in 3–11.6%, and nausea and vomiting in 7–10%.

Reported bradycardia was managed with atropine. Some control groups consisted of no adjunctive therapy while others consisted of an adjunctive opioid instead of dexmedetomidine. Although definitions of adverse events were consistent across trials, observed incidence varied. This likely reflects sample size differences and small cohort sizes, in which the absence or occurrence of events significantly impacts percentage estimates. Between-study variability should be interpreted cautiously.

In available obstetric epidural studies, reported sedation scores often fall in the range of 2 to 3 on the Ramsay Sedation Scale, corresponding to patients who are awake, calm, and responsive to verbal stimuli [56]. This level of sedation allows continued participation in labor [56]. Dexmedetomidine sedation is dose dependent. At obstetric dosing regimens, sedation is generally mild, and excessive drowsiness has been infrequently reported [37,57]. Overall, maternal sedation appears mild, dose-dependent, and neither associated with the loss of protective airway reflexes nor impaired maternal labor participation.

### 6.2. Neonatal Safety

Neonatal safety was evaluated using neonatal Apgar scores assessed at 1 and 5 min, umbilical cord blood gas analysis (including pH), and fetal heart rate. None of the studies reported NICU admission or resuscitation. The absence of reported NICU admissions or resuscitation events may reflect true low event rates. However, inconsistent reporting across studies limits definitive conclusions. Across included studies, no clinically significant differences in 1- or 5 min Apgar scores were observed between groups. Fetal bradycardia ranged from 0–11.3% in dexmedetomidine groups and from 0–8.6% in control groups. Of note, control groups across studies were non-uniform. Umbilical cord pH values remained within normal physiological ranges in the studies in which they were reported. Control groups were heterogeneous: some consisted of no adjunctive therapy while others consisted of an adjunctive opioid instead of dexmedetomidine. As with maternal outcomes, the relatively small sample sizes limit the ability to detect rare neonatal adverse effects. No fetal sedation indicators were reported in any of the included studies. 6.3 Rare Neuraxial Complications.

None of the studies included in this review reported neuraxial complications. Rare but serious neuraxial complications are unlikely to be captured in small randomized controlled trials and require large datasets for detection. Serious neuraxial complications include epidural hematoma, spinal hematoma, epidural abscess, and meningitis [58,59]. Meningitis after epidural anesthesia typically occurs a few days up to a week after epidural administration, which can complicate reporting time in smaller studies [60].

### 6.3. Practical Cautions for Off-Label Neuraxial Administration

Because dexmedetomidine is not FDA-approved as an adjuvant in epidural anesthesia, it is important to monitor for neurotoxicity signs and choose formulations without preservatives or compounded with additives. Neurotoxicity signs in general include paresthesia, motor dysfunction, chronic headaches, and sensory issues [61]. Preservative-containing adjuvants can lead to neurotoxicity when injected intrathecally, which may lead to arachnoiditis or meningitis in severe cases. For example, benzyl alcohol, a preservative used in local anesthetics, may cause flaccid paraplegia when used as part of the injectate that enters the epidural space [62]. While current preparations of local anesthetics have extremely low preservative concentrations, adjuvants may not be designed with this in mind. Therefore, it is imperative that clinicians use preservative-free or carefully monitor dexmedetomidine if used as an adjuvant in epidural anesthesia [63]. Finally, clinicians should ensure that all patients that receive adjuvant dexmedetomidine have informed consent. Clinicians should also disclose that dexmedetomidine is not FDA-approved for epidural anesthesia in any dose for the indication of labor analgesia. Clinicians should also inform patients of all relevant complications, especially those relating to neurotoxicity, and reiterate that the medication is being used off-label [64].

## 7. Fetal and Neonatal Safety

### 7.1. Placental Permeability

Dexmedetomidine can cross the placenta with moderate fetal exposure as demonstrated in cesarean delivery studies [65]. Reported umbilical vein-to-maternal vein ratios range from 0.68–0.76, indicating partial placental transfer [65,66]. Evidence of partial placental extraction and fetal redistribution, together with rapid maternal clearance, suggest that prolonged fetal exposure may be limited [65,66].

Drugs that cross the placenta have important implications, including reduced neonatal tone, respiratory drive, and alertness immediately after delivery [67]. In addition, placenta-crossing drugs can contribute to fetal heart rate changes indirectly through maternal physiological effects. Maternal hypotension following epidural analgesia or anesthesia may reduce uteroplacental perfusion and trigger non-reassuring fetal heart rates [55]. During labor, fetal effects observed after epidural anesthesia, such as fetal heart rate decelerations or bradycardia, are most often attributed to sympathetic blockade related hypotension or uterine hypotonus after analgesia [68].

### 7.2. Neonatal Outcomes

Available studies have not consistently demonstrated clinically significant adverse fetal heart rate changes or decreases in Apgar scores associated with dexmedetomidine when used as an epidural adjuvant [56]. Studies assessing dexmedetomidine in labor have typically assessed continuous electronic fetal heart monitoring and have reported no consistent increase in non-reassuring fetal heart rate tracing patterns [69]. Baseline fetal heart rate and variability were generally preserved, with no increase in late decelerations, persistent bradycardia, or category II or III tracings requiring intervention. Apgar scores were the most consistently reported neonatal outcome and were not significantly different between dexmedetomidine groups and opioid epidural comparator groups [56,65]. Other neonatal outcome measures, including umbilical cord blood gas analysis, neonatal oxygen saturation, and NICU admission, were not consistently reported across studies and therefore could not be compared [56,65].

## 8. Dexmedetomidine Versus Other Epidural Adjuvants

### 8.1. Dexmedetomidine vs. Clonidine

Dexmedetomidine demonstrates more profound anxiolytic effects compared to clonidine. In addition, epidural dexmedetomidine causes less hypotension than epidural clonidine [12].

### 8.2. Dexmedetomidine vs. Opioids

Dexmedetomidine has been associated with lower pain scores and improved analgesia quality in meta-analyses of labor studies [37]. Comparative trials evaluating dexmedetomidine against fentanyl or sufentanil generally report non-inferior or improved analgesia, often accompanied by reduced local anesthetic consumption in patient-controlled epidural analgesia [57,70]. Opioids adjuvants are classically associated with adverse effects such as pruritus, nausea, vomiting, urinary retention, and respiratory depression. In contrast, while dexmedetomidine is associated with fewer opioid-type side effects, it carries a potential risk for bradycardia when compared with epidural fentanyl [37,57]. Apgar scores and commonly reported neonatal outcomes are typically similar between dexmedetomidine and opioid adjuvant groups.

## 9. Discussion

To improve cross-study comparability, dexmedetomidine dosing was standardized and reported using either µg/kg or µg/kg/h for bolus and continuous infusion, respectively. Several conclusions appear robust across dosing strategies: decreased VAS scores, increased duration of analgesia, increased incidence of bradycardia, and decreased time to analgesia.

In contrast, effects on hypotension showed inconsistent results. While superiority over the control group was observed in some instances, most comparisons demonstrated insignificant differences, including the reported incidence of nausea, vomiting, and pruritus. Optimal dosing strategy and comparative effectiveness relative to other epidural anesthetic adjunctive therapies remain inconclusive based on the included studies.

Dexmedetomidine is an α_2_ adrenergic receptor agonist that decreases sympathetic outflow, modulating nociceptive pain transmission [16]. Dexmedetomidine is used as an adjunct therapy to increase the duration of analgesia in epidural anesthesia for labor analgesia [26,28]. Additionally, dexmedetomidine may have similar analgesic properties to opioids as an adjuvant to local anesthetics in available trials [29]. However, dexmedetomidine can cause hemodynamic instability, manifesting as maternal hypotension and bradycardia [37].

Additionally, dexmedetomidine does not appear to exacerbate nausea, vomiting, or pruritus when used in combination with opioids in available trials [11]. Dexmedetomidine was associated with lower VAS scores, incidence of pruritus, nausea, and vomiting. However, some studies found that dexmedetomidine decreased maternal HR [26]. Some studies found that dexmedetomidine decreases the duration of labor compared to local anesthetic alone [24]. However, there is no significant difference in duration of labor between opioids and dexmedetomidine [25,26,28]. Dexmedetomidine causes clinically insignificant maternal hypotension, and it has no effect on neonatal Apgar scores [24,25,28]. Based on available trials, dexmedetomidine appears to have the potential to be a safe and efficacious adjuvant to epidural anesthesia in parturients. Further studies are necessary to better understand the safety and efficacy of dexmedetomidine for epidural use during the different stages of labor.

## 10. Conclusions

Dexmedetomidine is a highly selective α_2_ agonist that may be associated with lower incidence of opioid-typical side effects, such as pruritus, compared with opioids in available trials. Dexmedetomidine does not appear to cause a significantly increased motor blockade. Additionally, dexmedetomidine does not appear to prolong the duration of labor based on available trials. While dexmedetomidine appears to be an epidural anesthesia adjuvant with fewer opioid-associated side effects such as pruritus, its use in epidural anesthesia for labor analgesia is not FDA-approved. More clinical trials that directly compare the efficacy of dexmedetomidine to other α_2_ agonists and opioids in labor analgesia as an epidural anesthetic are needed, as well as more studies that evaluate the efficacy and safety of epidural dexmedetomidine during the different stages of labor. Several limits should be noted. The studies investigated in this narrative review are limited in their generalizability because all are single-center studies that excluded women with hemodynamic instability. The exclusion of women with underlying hemodynamic instability introduced selection bias. Not all relevant variables were available for all 5 studies (for example, fetal hypotension). Unmeasured confounders, such as socioeconomic factors, could have influenced the results of the studies used. Therefore, it is unclear whether the decrease in side effects in dexmedetomidine compared to the other medication, either an opioid or no adjuvant, can be generalized to women who do have underlying health conditions that include baseline hemodynamic instability. Future clinical trials should include a more robust assessment of neonatal safety beyond Apgar scores and umbilical artery pH. Fetal heart rate, Apgar scores, NICU admission, and umbilical artery blood gas analysis and pH should be assessed in each future trial to decrease the heterogeneity of the design of each study. Future studies should also include women with underlying hemodynamic instability provided there are no absolute contraindications to dexmedetomidine or epidural anesthesia. Importantly, no neuraxial complications were reported in the 5 studies used in this clinical review. Rare but serious neuraxial complications are unlikely to be captured in small randomized controlled trials.

## Data Availability

No new data were created or analyzed in this study.
